# Prevalence of a Novel Bunyavirus in Tea Tussock Moth *Euproctis pseudoconspersa* (Lepidoptera: Lymantriidae)

**DOI:** 10.1093/jisesa/ieab045

**Published:** 2021-07-19

**Authors:** Xiaoqing Wang, Qiaoying Gu, Wei Zhang, Hongyan Jiang, Shichun Chen, Guy Smagghe, Jinzhi Niu, Jin-Jun Wang

**Affiliations:** 1 Key Laboratory of Entomology and Pest Control Engineering, College of Plant Protection, Southwest University, Chongqing, China; 2 Tea Research Institute of Chongqing Academy of Agricultural Science, Chongqing, China; 3 International Joint Laboratory on China-Belgium Sustainable Crop Pest Control, Academy of Agricultural Sciences, Southwest University, Chongqing, China

**Keywords:** Phenuivirida, viral small RNA, replication strand, tea pests

## Abstract

*Euproctis pseudoconspersa* is a major pest of tea plants, and also causes a skin rash on workers in tea plantations. Research on virus could provide fundamental insights for classification, genetic diversity, evolution, and host–virus interaction mechanisms. Here, we identified a novel RNA virus, *Euproctis pseudoconspersa bunyavirus* (Phenuiviridae), and found that it is widely distributed in field populations of *E. pseudoconspersa*. The replication of virus in *E. pseudoconspersa* was indicated by Tag-PCR. These results contribute to the classification of bunyaviruses and provide insight into the diversity of commensal *E. pseudoconspersa bunyavirus* and the host.

The tea tussock moth, *Euproctis pseudoconspersa* (Strand) (Lepidoptera: Lymantriidae), is one of the major insect pests of tea plants. The larva of this insect pest can also cause allergic reaction on human skin (Wang et al. 2005). Besides chemical control of this pest, environmentally friendly pest control approaches are normally used to reduce insecticide residues in tea products, for instance, nucleopolyhedrovirus (Tang et al. 2009). More recently, the development of high-throughput sequencing technologies has facilitated the discovery of other virus species with no obvious pathological signs of infection. RNA viruses are increasingly reported in various invertebrate species (Shi et al. 2016, Niu et al. 2019, Zhang et al. 2019). Some viruses can not cause pathology that are isolated from asymptomatic insect hosts, which have been reported to be beneficial to their hosts (Xu et al. 2014, Parry et al. 2020).

In this study, a virus-related sequence was discovered in a field population of *E. pseudoconspersa* by high-throughput sequencing. Alignment and phylogenetic analyses of this potential virus revealed that it is a member of the bunyavirus group in the family Phenuiviridae. This virus has a negative polarity, single-stranded RNA comprising three segments: large (L), medium (M), and small (S). Thus, we tentatively named this novel virus as *Euproctis pseudoconspersa bunyavirus* (EpBYV). Detection of EpBYV by RT-PCR and Sanger-sequencing revealed that it is widely distributed among various field collected populations. In addition, the replication of this virus in tea tussock moths was implied by tag-PCR. These results suggested high level of prevalence of EpBYV in wild population and asymptomatic in the hosts. The actual effect of EpBYV to this host still requires further investigation.

## Materials and Methods

### Insect Collection, RNA Preparation, and Library Construction

Tea tussock moths were collected from tea orchards in July 2017 in Qingyuan district of Guangdong province, China (Fig. 2A). Total RNA was extracted from five larvae using TRIzol reagent (Invitrogen, Carlsbad, CA). Two libraries were constructed, and the RNA-seq data were screened to identify potential virus-related sequences. After removal of rRNA using a TruSeq Total RNA Sample Prep Kit (Illumina, San Diego, CA), RNA was sequenced on the Illumina HiSeq X Ten platform (paired-end reads of 150 bp), generating ~8 Gbp raw data (NCBI-SRR10428075). Then, a small RNA library was constructed to identify potential virus-derived small RNAs. The library of small RNAs of 18–30 nt with PE50 was prepared with the NEB Next Ultra small RNA Sample Library Prep Kit (Illumina) and sequenced by the HiSeq X Ten platform, generating ~10 Mb raw data (NCBI-SRR10439009).

### Analyses of Virus-related Sequence and Virus-derived Small RNAs

Raw reads from the RNA-seq library were analyzed by CLC Genomics Workbench 9.5 (Qiagen, Hilden, Germany). After adaptor trimming and filtering of low-quality reads, de novo assembly of clean reads was conducted using Trinity software (RNA). The assembled contigs were subjected to BLASTx and BLASTn searches of viral sequences in GenBank databases. Viral ORFs were predicted using the online tool Gene Finding in Viral Genomes. Conserved and functional domains were identified by searches at the Conserved Domain Database in NCBI and by using the SMART tool. The phylogenetic tree was constructed using the RNA-dependent RNA polymerase (RdRP) amino acid sequence. The tree was constructed with 1,000 bootstrap replicates with the Neighbor-joining method in MEGA 6.0 (Tamura et al. 2013). The small RNA clean data were mapped to virus-related sequences to identify virus-derived small RNAs using Bowtie2 software. These virus-derived small RNAs were further analyzed based on their length distribution and genomic positions on both positive and negative strands using the R package viRome (Watson et al. 2013).

### Viral Detection in Field Populations

To investigate the prevalence of the virus in field populations of *E. pseudoconspersa* in the main tea cultivation areas of China (Cao et al. 2019), we collected larval samples from filed. The larvae of tea tussock moth normally cluster together in tea plants, in order to avoid the bias of this behavior in sample collections, we tested 20 larvae in each District per year (each larval individually as a sample; only 2–3 larvae were randomly selected from each larval cluster). In total, we tested a number of 220 larvae from all of the Districts from 2017 to 2019. The sampling areas included Yongchuan District, Wanzhou District, and Nanchuan District in Chongqing City, Xinyang City in Henan province, Wuxi City in Jiangsu province, Anqing City in Anhui province, and Guilin City in Guangxi province (Fig. 2A). Two kinds of PCR were performed: (1) RT-PCR for the presence of virus. RNA was synthesized to cDNA via PrimerScriptRT Reagent Kit (TaKaRa, Dalian, China) oligo(dT) and random primers. The samples were analyzed by RT-PCR with the primer pair Bunyavirus-F (5′-GAGGCCCAATG-TATCTGGCT-3′) and Bunyavirus-R (5′-ACTGTGGGAAGAGTGTTGTGT-3′) and the following thermal cycling program: 95°C for 3 min, 40 cycles at 95°C for 30 s, 60°C for 30 s and 72°C for 1 min, and final extension at 72°C for 5 min. We counted the frequency of the virus in tea tussock moth by the ratio of the positive samples to the 20 tested samples. (2) Tag-PCR for detection of viral replication. A non-homologous tag sequence (5′-TCGAGTGTCACAGTCACGAC-3′) can be added to the primer used in the reverse-transcription with the subsequent PCR then using this tag sequence as a primer in combination with the viral-specific primer (replication strand). Reverse transcription can also be undertaken using a thermostable reverse transcriptase at elevated temperatures to further reduce the likelihood of false priming. After the treatment of exonuclease to eliminate the primer, the synthesized cDNA was then used as template for Tag-PCR, thereby further reducing the chance of false positives. Next, the Tag-PCR was performed by the forward primer Tag and the reverse primer (virus-specific sequence). Twelve virus-positive samples (determined by (1) RT-PCR) from four groups (three samples per group) were randomly chosen for the detection of the replication strand (positive sense of this virus) by Tag-PCR (Highfield et al. 2020). Briefly, 500 ng RNA was reverse transcribed using the tag- Bunyavirus primer (5′-TCGAGTGTCACAGTCACGAC-CGCCTGAGAGGTGTGCTATT-3′) at 55°C. An amount of 1 µl this cDNA was taken as template by using primers tagF (5′-TCGAGTGTCACAGTCACGAC-3′) and Bunyavirus-R (5′-CGAGCTGACAGTAGGGTTCC-3′). Reactions were undertaken in 25-µl volumes and were incubated at 95°C for 2 min, followed by 40 cycles at 95°C for 5 s, 57°C for 10 s, and 72°C for 10 s. Both the products of RT-PCR (for virus detection) and Tag-PCR (for viral replication strand detection) were confirmed by Sanger sequencing.

### Ethical Approval

This article does not contain any studies with human participants or animals performed by any of the authors.

## Results and Discussion

### A Novel Bunyavirus

**Fig. 1.**
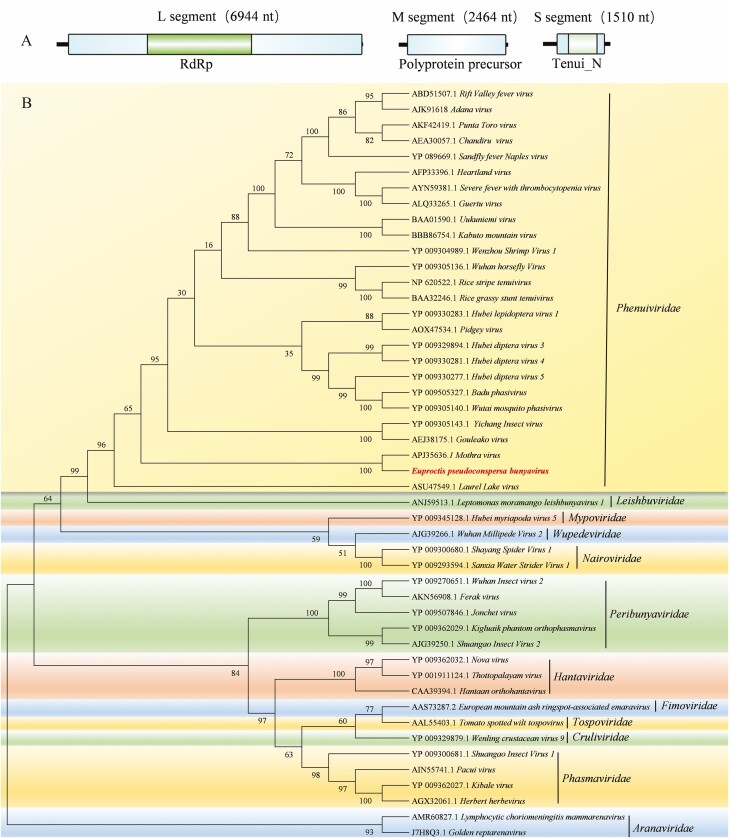
(A) Genome organization of *Euproctis pseudoconspersa bunyavirus*. Genome consists of L, M, and S segments. One ORF in L segment encodes a protein annotated as an RNA-dependent RNA polymerase (RdRp). An ORF in M segment encodes a protein annotated as a glycoprotein precursor by Blastp. S segment has one ORF encoding a protein with conserved domains typical of Tenui_N superfamily. (B) Phylogenetic tree of *Euproctis pseudoconspersa bunyavirus* constructed using amino acid sequences of RdRp (generated with 1,000 replicates using Muscle alignment and Neighbor-joining method).

One virus-related sequence (10,918 nt) was identified from the constructed RNA-seq library. This sequence contained three segments (L, M, and S). In the L segment, the ORF from nucleotide position 269 nt to 6940 nt encoded an RdRp (NCBI-MN752229). In the M segment, the nt sequence 190–242 encoded a protein of 744 aa that was annotated as a glycoprotein precursor (NCBI-MN752230). This protein showed identities of 24.6% with *Wuchang Cockroach virus 1* (family Phasmaviridae), 23.9% with *Anopheles triannulatus orthophasmavirus* (family Phasmaviridae), and 23.5% with *Mothra virus* (family Phenuiviridae). The S segment contained one ORF (nt 308–1387) encoding a 359-aa protein with conserved domains typical of proteins in the Tenui_N superfamily (NCBI-MN752231). This protein, which was identified as a nucleocapsid protein, showed similarities to nucleocapsid proteins of the genera *Tenuivirus* (family Phenuiviridae) and *Phlebovirus* (family Phenuiviridae) with E-value of 5.2 e-10, but differed from those of other members of the *Bunyavirales* (Fig. 1A). In the phylogenetic analysis, this virus clustered into the family *Phenuiviridae*, and its sequences showed similarities to those of the *Mothra virus* that infects invertebrates (44.97%, 34.13%, and 37.36% similarities of the L, M, and S segment, respectively; Fig. 1B; Abudurexiti et al. 2019). Thus, we tentatively named this novel virus *Euproctis pseudoconspersa bunyavirus* (EpBYV). A bunyavirus in lepidopteran insects has also been reported, namely Hubei lepidoptera virus 1 (NCBI-LCM141331) (Shi et al. 2016).

We also explored the viral-derived small RNAs from EpBYV with parallel small RNA sequencing. A library with a 10 M dataset of small RNAs was obtained, but none of these small RNAs were mapped to the genome of EpBYV. In our previous study, we identified a signal of a 22-nt virus-derived small RNA, suggesting that host RNAi is involved in the defense response to bunyavirus infection in aphids. We postulate that the infection of EpBYV may not necessarily induce the response of RNAi as antiviral immunity in *E. pseudoconspersa*, since this could be determined by viral infection stage, viral titters, host immunity hemostasis, and so on, thus further studies are required to analyze the immune response of the hosts in infection of EpBYV.

### Prevalence of Virus in Field Populations

**Fig. 2.**
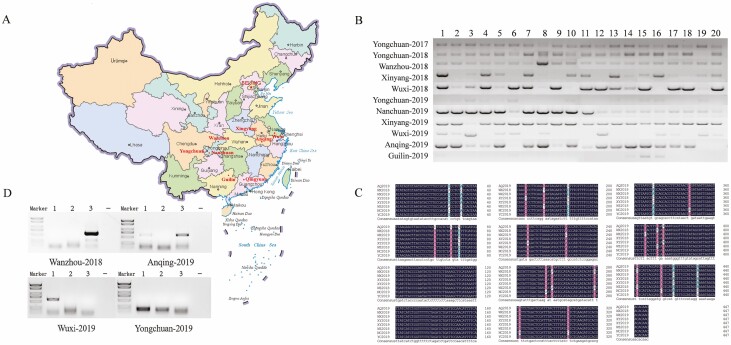
(A) Collection sites of Tea tussock moth in different areas in China. Tea orchards are marked in red. (B) Detection of *Euproctis pseudoconspersa bunyavirus* in Tea tussock moth samples (*n* = 20) collected from different tea plantations 1–20 representing collected samples(C) Analysis of partial RdRp mutations of EpBYV in each population. (D) Detection of the replicative strand in four populations of Tea tussock moth by Tag-PCR. Three samples (EpBYV positive were selected by PCR of Fig. 2B) and one negative control (water served as control) were performed in each population (Wanzhou-2018, Anqing-2019, Wuxi-2019, and Yongchuan-2019).

The detection rate of EpBYV was the highest in the Wanzhou-2018 and Nanchuan-2019 samples (100.0%), followed by Yongchuan-2017 (95.0%), Yongchuan-2018 (95.0%), Xinyang-2019 (95.0%), Anqing-2019 (85%), Wuxi-2018 (80%), Xingyang-2018 (70%), Wuxi-2019 (55%), Yongchuan-2019 (45.0%), and Guilin-2019 (20%) (Fig. 2B). The sequenced PCR products from the above analyses revealed 24 sites with potential single nucleotide polymorphisms that did not result in amino acid mutations (Fig. 2C). These results showed that EpBYV were prevalence in field populations of *E. pseudoconspersa*, and the high infection rate of this virus pointed out an existed interaction between the virus and the host. Secondly, by using the above viral positive samples, we detected the viral replication strand by Tag-PCR. The results showed that 4 out of 12 samples presented the replication strand of EpBYV, which were Wanzhou-2018 (sample 3), Anqing-2019 (samples 1 and 3) and Wuxi-2019 (sample 1), respectively (Fig. 2D). Taken together, these findings show that this newly identified virus is widely distributed among field populations of *E. pseudoconspersa* and potentially replicate among some hosts.
